# Associations Between Excessive Sodium Intake and Smoking and Alcohol Intake Among Korean Men: KNHANES V

**DOI:** 10.3390/ijerph121215001

**Published:** 2015-12-08

**Authors:** Kyung-Hwa Choi, Myung-Sook Park, Jung Ae Kim, Ji-Ae Lim

**Affiliations:** 1 Hallym Research Institute of Clinical Epidemiology, Hallym University, 1 Hallymdaehak-gil, Chuncheon, Gangwon-do 200-702, Korea; rosach72@hanmail.net; 2 Taean Institute of Environmental Health Center, 1952-16 Seohaero, Taean-eup, Taean-gun, Chungcheongnam-do 32148, Korea; pms1816@gmail.com; 3 Department of Social and Preventive Medicine, College of Medicine, Hallym University, 1 Hallymdaehak-gil, Chuncheon, Gangwon-do 200-702, Korea; sokja@hallym.ac.kr; 4 College of Nursing, Hanzhong University, 200 Jiyang-gil, Donghae, Gangwon-do 240-713, Korea; 5 Department of Preventive Medicine, College of Medicine, Dankook University, 119 Dandae-ro, Cheonan, Chungnam 330-714, Korea

**Keywords:** smoking, alcohol intake, excessive sodium intake, KNHANES

## Abstract

In this study, we evaluated the associations of smoking and alcohol intake, both independently and collectively, with sodium intake in Korean men. Subjects (6340 men) were from the fifth Korean National Health Examination Survey (2010–2012). Smoking-related factors included smoking status, urinary cotinine level, and pack-years of smoking. Food intake was assessed using a 24-h recall. The odds of excessive sodium intake were estimated using survey logistic regression analysis. The smoking rate was 44.1%. The geometric mean of the urinary cotinine level was 0.05 µg/mL, and the median (min–max) pack-years of smoking was 13.2 (0–180). When adjusted for related factors, the odds (95% confidence interval) of excessive sodium intake were 1.54 (1.00, 2.37), 1.55 (1.23, 1.94), 1.44 (1.07, 1.95), and 1.37 (1.11, 1.68) times higher in the group exposed to smoking and drinking than in the group that never smoked nor drank, the group that never smoked and drank <5 times per month, the group that did not currently smoke and never drank, and the group that did not currently smoke or drink <5 times per month, respectively. There was an interaction effect between smoking and alcohol intake (*p-interaction* = 0.02). The results suggest that simultaneous exposure to smoking and alcohol intake is associated with increased odds of excessive sodium intake.

## 1. Introduction

According to the Korean National Health Examination Survey (KNHANES), the smoking rate in Korea has consistently decreased among men aged ≥19 years, from 66.3% in 1998 to 42.1% in 2013 [1]. However, among OECD countries, the rate of smoking in Korea still ranks second, after Greece, for men aged ≥15 years [2]. Moreover, the starting age for smoking has decreased, from 14.1 years in 2005 to 13.9 years in 2010 and 13.7 years in 2014 [3]. 

Smoking has negative effects on psychological conditions such as depression [4] and increases the risk of arteriosclerosis, heart disease, and respiratory diseases such as asthma and chronic obstructive pulmonary disease [5]. It is the main cause of laryngeal, oral, pancreatic, and, in particular, lung cancers [6] and premature death [7]. It also affects taste perception [8,9], consistently dulling the perception of bitter [10] and sour tastes in current smokers [11], and smokers might have a greater preference for salty and sweet tastes than non-smokers [12].

Alcohol intake also adversely affects health, particularly when coupled with smoking, and smokers tend to have a higher alcohol intake than non-smokers [13,14]. A study based on the Korean Community Health Survey 2008 reported a negative association between a low-sodium diet and smoking and alcohol intake [15]. Furthermore, using the KNHANES 2010, the proportion of subjects with a sodium intake >4000 mg/day was reportedly higher in smoking and drinking groups [16].

Sodium is an essential element in the human body, and the daily dietary requirement of sodium is 1500 mg [17]. The daily sodium intake in Korea has steadily decreased, from 5861 mg in 2005 to 5597 mg in 2010 and 4659 mg in 2013. However, it remains more than twice that recommended by the World Health Organization (2000 mg) [1]. Excessive sodium intake is a major predisposing factor for increased blood pressure [18], stroke, and heart disease [19]. It also increases the risk of stomach cancer and is a risk factor for osteoporosis due to excretion of calcium from the bones [20]. 

Although the health effects of excessive sodium intake and unhealthy behaviors due to smoking and alcohol intake have been reported [15,16], few studies have evaluated the association between smoking and sodium intake in the general population and the combined effect, if any, of smoking and alcohol intake on sodium intake. 

Therefore, this study aimed to evaluate the association of smoking and alcohol intake with excessive sodium intake among men using data from KNHANES V.

## 2. Materials and Methods 

### 2.1. Study Subjects

Data were derived from the fifth KNHANES (2010–2012). The KNHANES is a series of national health surveys in Korea that use a stratified multistage probability sampling design to select a representative sampling of the Korean population. The KNHANES V health interview surveys were conducted through face-to-face interviews by trained interviewers at the homes of subjects. Informed consent was provided by each subject prior to inclusion in the study [21]. 

**Figure 1:**
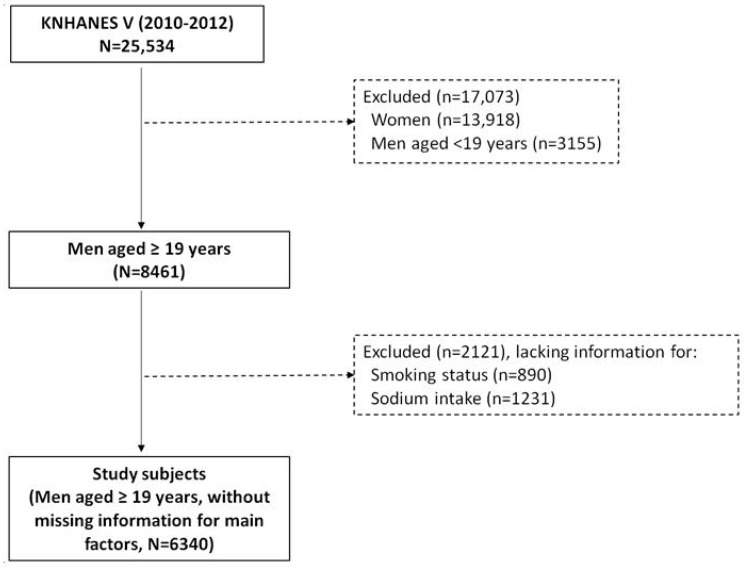
Selection process of study subjects, KNHANES V (2010–2012).

We included only men because they are the main consumer of tobacco, and women tend to underreport their smoking status due to social and cultural characteristics in Korea [22]. Initial candidates for this study were 25,534 subjects; of these, 13,918 women of any age and 3155 men aged <19 years were excluded. Further, 890 and 1231 subjects lacking information for smoking or sodium intake, respectively, were excluded. Finally, 6340 subjects were included (Figure 1).

### 2.2. Smoking-Related Factors

Smoking-related factors included smoking status, urinary cotinine level, and pack-years of smoking. Smoking status was determined using the questionnaire and categorized as non-smoker, ex-smoker, or current smoker (including smoking often). Urinary cotinine levels were analyzed in 2010 and 2011 and measured using gas chromatography and mass spectrometry with a Perkin Elmer Clarus 600T (PerkinElmer, Turku, Finland), cotinine (Sigma, St. Louis, MO, USA), and diphenylamine (Aldrich, St. Louis, MO, USA) (threshold of detection, 0.25 ng/mL) [21]. We corrected for urine dilution by using urinary creatinine concentrations and calculated creatinine-standardized cotinine concentrations (*vs*. non-standardized) by dividing individual urinary cotinine concentrations by creatinine concentrations. Pack-years of smoking (Equation (1)) were calculated using information collected with the questionnaire and the following formula:

(1) $$Pack-years\;of\;smoking=\frac{Number\;of\;cigarettes\;smoked\;per\;day}{20\;\left(the\;number\;of\;cigarettes\;in\;a\;pack\right)}\times the\;number\;of\;year\;smoked$$

### 2.3. Sodium Intake

Food intake was assessed using a 24-h recall. Trained interviewers visited each subject’s house to collect food name, ingredients, and amount of intake during the previous 24 h. Data were converted to type and amount of food items in the form of units of food or nutrients, because the form of intake is a combination of foods. The reference food composition tables were from the seventh edition of the Korean National Rural Living Science Institute [23]. The Korea Health Industry Development Institute database was also used for some instant foods and imported foods [24]. Excessive sodium intake was defined as intake greater than the third quartile of sodium intake (>7392.52 mg/day).

### 2.4. Urinary Creatinine Level

Urinary creatinine level was analyzed in 2010 and 2012 and measured using a Creatinine-HR 1-Type Wako (WAKO, Osaka, Japan) with a Hitachi Automatic Analyzer 7600 (Hitachi, Tokyo, Japan) and colorimetry [21]. 

### 2.5. Confounding Factors

Age (19–34, 35–64, and ≥65 years), household income (low, lower-middle, upper-middle, and high), marital status (married; separated, widowed, or divorced; and never married), frequency of alcohol intake per month (none, 0–1, 2–4, and ≥5), and frequency of eating out (<1/month, 1–8/month, 3–6/week, and ≥1/day) were collected using the KNHANES questionnaire. Weight and height were measured by the KNHANES team to calculate body mass index (BMI), and we categorized participants as underweight (<18.5 kg/m^2^), normal weight (18.5–24.9 kg/m^2^), or overweight (≥25 kg/m^2^). When we analyzed non-standardized cotinine level, log transformed creatinine level was used for confounding factor. These confounding factors were previously used to estimate the risk of smoking and excessive sodium intake [15,16]. Among the socioeconomic status factors, household income and educational level were evaluated. Only household income was used in the final analyses because the associations between household income and smoking factors were greater than those with educational level.

### 2.6. Statistical Analysis

Survey chi-square tests were performed to evaluate the distribution of smoking status according to general characteristics. We used a weighted population sample to reflect the sampling method and response rate. Survey regression analysis was performed to assess the distribution of urinary cotinine levels, pack-years of smoking, and sodium intake according to general characteristics. Spearman correlation tests were performed to evaluate the correlations among the exposure variables. Odds ratios (ORs) and 95% confidence intervals (CIs) for excessive sodium intake were estimated using survey logistic regression analysis. 

The *p*-trend was estimated using a continuous scale for each category. The *p*-interaction was estimated as the *p*-value of the interaction term between smoking status and alcohol intake. Missing values were excluded from the analyses with the general characteristics. Missing values were included in an “Unknown” category when variables with missing values were used as adjusting factors. All analyses were performed using SAS 9.4 [25], and significance was set at 0.05.

## 3. Results 

### 3.1. Distribution of Smoking-Related Factors

Table 1 describes the distribution of smoking-related factors according to the general characteristics. 

**Table 1:** Distribution of smoking-related factors according to general characteristics among men aged ≥19 years in KNHANES V, 2010–2012.

	All	Current Smoker		Urinary Cotinine Level (μg/mL)	Pack-Years of Smoking
	N	N	% ^w^ (SE)	GM ^a^ (95% CI)	Median (min–max)
All	6340	2450	44.1 (0.8)	0.05 (0.04, 0.07)	13.2 (0–180)
Age group (years)					
19–34	1071	507	47.9 (1.6)	0.06 (0.04, 0.08)	2.4 (0–33)
35–64	3545	1528	46.6 (1.0)	0.06 (0.04, 0.07)	15.8 (0–156)
≥65	1724	415	25.1 (1.2)	0.02 (0.01, 0.03)	20.0 (0–180)
*p*-value			<0.0001	0.0003	<0.0001
Household income					
Low	1202	439	42.7 (2.1)	0.1 (0.06, 0.16)	20.0 (0–156)
Lower middle	1631	659	46.8 (1.7)	0.07 (0.04, 0.10)	13.5 (0–180)
Upper middle	1744	681	44.9 (1.4)	0.05 (0.03, 0.07)	10.5 (0–129)
High	1705	647	41.3 (1.6)	0.04 (0.03, 0.06)	10.3 (0–180)
Unknown	58	58	47.8 (9.0)	0.07 (0.00, 2.96)	16.4 (0–85.5)
*p*-value			0.01	0.03	<0.0001
Marital status					
Married	5109	1868	42.1 (0.9)	0.04 (0.03, 0.06)	15.1 (0–180)
Separated, widowed, or divorced	314	147	54.2 (3.6)	0.21 (0.08, 0.53)	23.9 (0–180)
Not married	912	432	47.8 (1.8)	0.07 (0.05, 0.10)	2.0 (0–74)
Unknown	5	3	58.3 (25.6)	-	40.0 (12.9–86)
*p*-value			<0.0001	0.001	<0.0001
Frequency of alcohol intake (/month)					
None	1098	244	24.1 (1.7)	0.02 (0.01, 0.03)	15.7 (0–147)
0–1	1215	405	37.8 (1.8)	0.03 (0.02, 0.05)	8.3 (0–180)
2–4	1666	675	45.1 (1.5)	0.05 (0.03, 0.07)	8.9 (0–150)
≥5	2333	1116	54.2 (1.3)	0.11 (0.08, 0.15)	18.5 (0–180)
Unknown	28	10	46.6 (8.8)	0.02 (0.00, 0.19)	7.5 (0–87.5)
*p*-value			<0.0001	<0.0001	<0.0001
Body mass index (kg/m^2^)					
<18.5	192	92	51.4 (4.8)	0.04 (0.01, 0.32)	17.0 (0–156)
18.5 to <25	3940	1538	43.8 (1.0)	0.05 (0.04, 0.07)	12.8 (0–180)
≥25	2182	807	44.1 (1.3)	0.06 (0.04, 0.08)	14.0 (0–180)
Unknown	26	13	44.7 (11.5)	0.01 (0, 6203.2)	10.5 (0–45)
*p*-value			0.14	0.93	0.006
Frequency of eating out					
<1/month	646	214	35.4 (2.5)	0.04 (0.01, 0.11)	24.8 (0–156)
1–8/month	2131	712	39.4 (1.5)	0.06 (0.04, 0.08)	17.5 (0–180)
3–6/week	1615	633	45.4 (1.5)	0.05 (0.03, 0.07)	10.0 (0–180)
≥1/day	1946	890	48.5 (1.4)	0.06 (0.04, 0.08)	10.0 (0–140)
Unknown	2	1	42.6 (35.0)	-	8.5 (0.5–16.5)
*p*-value			<0.0001	0.70	<0.0001
Correlation coefficient with urinary cotinine level (*p*-value)			0.63 (<0.001)	-	0.27 (<0.001)

^a^ SE: standard error; GM: geometric mean; CI: confidence interval; *p*-value estimated using survey chi-square tests or survey regression analyses.

The smoking rate was 44.1%. The geometric mean (95% CI) of the urinary cotinine level was 0.05 (0.04, 0.07) µg/mL, and the median (min–max) pack-years of smoking was 13.2 (0–180). Smoking rates and urinary cotinine levels were higher with younger age and more frequent alcohol intake. The factors associated with smoking rate and urinary cotinine level, in increasing order of effect, were lower-middle income, marital separation, and obesity. Pack-years of smoking were higher with older age, rarely eating out, and low income. Marital separation and underweight were related with pack-years of smoking. The correlation between smoking status and urinary cotinine level (ρ = 0.63, *p* < 0.001) was higher than that between pack-years of smoking and urinary cotinine level (ρ = 0.27, *p* < 0.001).

### 3.2. Distribution of Sodium Intake 

Table 2 describes the distribution of sodium intake according to the general characteristics. The median sodium intake was significantly different across the distributions of all of the characteristics. Sodium intake was significantly lower in the older and marital separation groups. It was higher with increasing income, frequency of alcohol intake, BMI, and frequency of eating out. 

**Table 2:** Distribution of sodium intake (mg/day) according to general characteristics among men aged ≥19 years in KNHANES V, 2010–2012.

	All	Sodium Intake (mg/day)
	N	GM ^a^ (95% CI)
All	6340	5133.2 (5034.5, 5233.9)
Age group (years)		
19–34	1071	5170.7 (4967.5, 5382.2)
35–64	3545	5432.3 (5310.6, 5556.8)
≥65	1724	3955.6 (3802.2, 4115.1)
*p*-value		<0.0001
Household income		
Low	1202	4253.6 (4047.6, 4470.1)
Lower middle	1631	5050.2 (4868.3, 5238.8)
Upper middle	1744	5409.6 (5242.9, 5581.7)
High	1705	5442.4 (5232.8, 5660.4)
Unknown	58	4707.9 (4111.7, 5390.4)
*p*-value		<0.0001
Marital status		
Married	5109	5239.5 (5132.6, 5348.6)
Separated, widowed, or divorced	314	4743.3 (4346.6, 5176.3)
Not married	912	4920.9 (4712.2, 5138.9)
Unknown	5	-
*p*-value		0.01
Frequency of alcohol intake (/month)		
None	1098	4547.9 (4324.1, 4783.4)
0–1	1215	4900.7 (4699.0, 5111.1)
2–4	1666	5195.0 (5013.7, 5382.7)
≥5	2333	5450.9 (5297.4, 5609.0)
Unknown	28	5797.3 (4817.5, 6976.4)
*p*-value		<0.0001
Body mass index (kg/m^2^)		
<18.5	192	4540.5 (4119.9, 5004.0)
18.5 to <25	3940	4952.6 (4835.7, 5072.2)
≥25	2182	5510.2 (5342.6, 5683.1)
Unknown	26	4834.9 (3718.6, 6286.3)
*p*-value		<0.0001
Frequency of eating out		
<1/month	646	3906.3 (3622.5, 4212.3)
1–8 month	2131	4636.1 (4474.7, 4803.2)
3–6/week	1615	5397.2 (5213.6, 5587.1)
≥1/day	1946	5637.5 (5463.5, 5817.1)
Unknown	2	6665.4 (29.7, 1,498,256.3)
*p*-value		<0.0001

GM: geometric mean; CI: confidence interval; *p*-value estimated using survey regression analysis.

### 3.3. Odds of Excessive Sodium Intake According to Smoking-Related Factors 

Table 3 describes the odds for excessive sodium intake according to the smoking-related factors. The odds were higher for current smokers and the group with the highest cotinine levels, but this was not statistically significant when adjusted for related factors. 

**Table 3:** Odds ratios (ORs) and 95% confidence intervals (CIs) for excessive sodium intake according to smoking-related factors among men aged ≥19 years in KNHANES V, 2010–2012.

Smoking-Related Factors	Excessive Sodium Intake (>7392.52 mg/day) ^a^			
			Crude Model	Adjusted Model ^b^
	N	Cases	OR (95% CI)	OR (95% CI)
Smoking status				
Non-smoker	1176	264	1.00 (ref)	1.00 (ref)
Ex-smoker	2714	666	1.21 (1.00, 1.47)	1.16 (0.94, 1.43)
Current smoker	2450	655	1.33 (1.10, 1.61)	1.17 (0.95, 1.43)
*p-trend*			*0.01*	*0.22*
Urinary cotinine level (μg/mL)				
Q1 (0.009, 3.07)	387	112	1.00 (ref)	1.00 (ref) ^c^
Q2 (3.07, 14.5)	387	104	0.82 (0.56, 1.21)	0.77 (0.52, 1.14)
Q3 (14.5, 1133.38)	388	110	0.99 (0.70, 1.41)	0.96 (0.66, 1.40)
Q4 (1133.38, 5800.17)	387	137	1.31 (0.93, 1.84)	1.29 (0.89, 1.86)
*p-trend*			*0.05*	*0.08*
Urinary cotinine level corrected by creatinine level (μg/g Cr)				
Q1 (0.00002, 0.02)	387	106	1.00 (ref)	1.00 (ref)
Q2 (0.02, 0.11)	387	108	0.85 (0.57, 1.27)	0.83 (0.55, 1.26)
Q3 (0.11, 6.63)	388	112	1.10 (0.76, 1.57)	1.07 (0.73, 1.56)
Q4 (6.63, 63.78)	387	137	1.34 (0.94, 1.90)	1.26 (0.87, 1.83)
*p-trend*			*0.04*	*0.11*
Pack-years of smoking				
Q1 (0, 1.3)	1531	350	1.00 (ref)	1.00 (ref)
Q2 (1.3, 13)	1517	400	1.28 (1.05, 1.56)	1.15 (0.94, 1.41)
Q3 (13, 28)	1539	423	1.29 (1.06, 1.58)	1.10 (0.88, 1.38)
Q4 (28, 180)	1511	342	1.09 (0.89, 1.32)	1.08 (0.86, 1.36)
*p-trend*			*0.27*	*0.53*

ORs and 95% CIs were estimated using survey logistic regression models. ^a^ Third quartile of sodium intake (mg/day) in KNHANES V; ^b^ Adjusted for age, household income, marital status, body mass index, frequency of alcohol intake, and frequency of eating out; ^c^ Additionally adjusted for log-transformed creatinine level. The *p*-trend was estimated using the continuous scale of each category in the same model.

### 3.4. Excessive Sodium Intake According to Alcohol Intake

Table 4 shows the odds for excessive sodium intake according to the frequency of alcohol intake. When adjusted for related factors, the odds of excessive sodium intake were 1.49 times (95% CI: 1.14, 1.95) higher in the group with an alcohol intake >5 times per month than in the non-alcohol intake group, and the OR increased with increasing frequency of alcohol intake per month (*p-trend* = 0.003). 

**Table 4:** Odds ratios (ORs) and 95% confidence intervals (CIs) for excessive sodium intake according to the frequency of alcohol intake per month among men aged ≥19 years in KNHANES V, 2010–2012.

	Excessive Sodium Intake (>7392.52 mg/day ^a^)			
	N	Cases	Crude model	Adjusted model ^b^
			OR (95% CI)	OR (95% CI)
Frequency of alcohol intake (/month)				
None	1098	190	1.00 (ref)	1.00 (ref)
0–1	1215	287	1.43 (1.08, 1.90)	1.25 (0.93, 1.67)
2–4	1666	425	1.43 (1.11, 1.85)	1.17 (0.90, 1.52)
≥5	2333	675	1.85 (1.43, 2.38)	1.49 (1.14, 1.95)
*p-trend*			*<0.0001*	*0.003*

ORs and 95% CIs were estimated using survey logistic regression models. ^a^ Third quartile of sodium intake (mg/day) in KNHANES V; ^b^ Adjusted for age, household income, marital status, body mass index, frequency of eating out, and smoking status. The *p*-trend was estimated using the continuous scale of each category in the same model.

### 3.5. Excessive Sodium Intake According to Combined Smoking and Alcohol Intake 

Table 5 shows the odds for excessive sodium intake according to combined smoking status and current alcohol intake. When adjusted for related factors, the odds (95% CI) for excessive sodium intake in the group exposed to both smoking and drinking were 1.54 times (1.00, 2.37), 1.55 times (1.23, 1.94), 1.44 times (1.07, 1.95), and 1.37 times (1.11, 1.68) higher than in the group that never smoked nor drank, the group that never smoked and drank <5 times per month, the group that did not currently smoke and never drank, and the group that did not currently smoke and drank <5 times per month, respectively. In addition, there was an interaction effect between smoking experience and alcohol intake (*p-interaction* = 0.02).

**Table 5:** Odds ratios (ORs) and 95% confidence intervals (CIs) for excessive sodium intake according to smoking status and alcohol intake among men aged ≥19 years in KNHANES V, 2010–2012.

		Risk Group of Excessive Sodium Intake (>7392.52 mg/day) ^a^			
		N	Case	Crude Model	Adjusted Model ^b^
				OR (95% CI)	OR (95% CI)
Smoking experience ^c^	Current alcohol intake				
No	No	279	46	1.00 (ref)	1.00 (ref)
No	Yes	893	217	1.39 (0.88, 2.18)	1.27 (0.80, 2.02)
Yes	No	819	144	1.08 (0.67, 1.72)	1.15 (0.71, 1.86)
Yes	Yes	4321	1170	1.76 (1.16, 2.67)	1.54 (1.00, 2.37)
*p-interaction*	**			*0.0005*	*0.02*
Smoking experience ^c^	Frequency of alcohol intake (≥5/month)				
No	No	912	190	1.00 (ref)	1.00 (ref)
No	Yes	260	73	1.45 (0.98, 2.15)	1.40 (0.94, 2.08)
Yes	No	3067	712	1.23 (0.99, 1.52)	1.22 (0.97, 1.53)
Yes	Yes	2073	602	1.64 (1.33, 2.02)	1.55 (1.23, 1.94)
*p-interaction*				*0.11*	*0.22*
Current smoker	Current alcohol intake				
No	No	854	145	1.00 (ref)	1.00 (ref)
No	Yes	3018	777	1.53 (1.16, 2.02)	1.30 (0.98, 1.74)
Yes	No	244	45	1.07 (0.66, 1.74)	1.00 (0.61, 1.63)
Yes	Yes	2196	610	1.75 (1.31, 2.33)	1.44 (1.07, 1.95)
*p-interaction*				*0.48*	*0.50*
Current smoker	Frequency of alcohol intake (≥5/month)				
No	No	2655	567	1.00 (ref)	1.00 (ref)
No	Yes	1217	355	1.54 (1.26, 1.87)	1.46 (1.20, 1.79)
Yes	No	1324	335	1.26 (1.04, 1.53)	1.19 (0.98, 1.46)
Yes	Yes	1116	320	1.5 (1.23, 1.83)	1.37 (1.11, 1.68)
*p-interaction*				*0.02*	*0.05*

ORs and 95% CIs were estimated using survey logistic regression models. ^a^ Third quartile of sodium intake (mg/day) in KNHANES V; ^b^ Adjusted for age, household income, marital status, body mass index, and frequency of eating out; ^c^ Smoking experience: no (never smoker) and yes (ex-smoker and current smoker). The *p-interaction* was estimated as the *p*-value of the interaction term between smoking status and alcohol intake, adjusted for the same confounding factors.

## 4. Discussion

We found that the combination of smoking and alcohol intake was associated with increased odds of excessive sodium intake. The results of the present study corroborate the findings of previous studies [26,27]; pack-years of smoking were higher in older men, but smoking rate and urinary cotinine levels were lower with increasing age. 

In the present study, smoking was not significantly associated with excessive sodium intake, which is similar to the results of a previous study using the fourth KNHANES in which sodium intake was much higher in light smokers than in non-smokers, but this was not significant [28]. However, our results differ from those of other studies [12,16]. Among middle-aged Korean workers, the proportion of current smokers with a sodium intake >4000 mg was higher than the proportion of non-smokers and past smokers [16]. Furthermore, in another study with subjects aged ≥18 years, the preference for saltier food was 1.5–2.3 times higher in smokers than in non-smokers [12]. 

In studies using a diet preference, smoking was associated with sodium intake [12,28]. However, in studies using a 24-h recall, the results were not significantly associated [28], as in the present study. Thus, the method of measuring sodium intake and the sample might yield varying results. In the present study, excessive sodium intake was significantly more likely with increasing frequency of alcohol intake, supporting the findings of previous studies [16,29]. The proportion of subjects with a sodium intake >4000 mg was higher in those who consumed alcohol [16], and a positive correlation between alcohol intake and sodium intake was reported among subjects aged ≥ 65 years [29]. Alcohol drinkers prefer saltier food 2.57–2.92 times more than non-drinkers [12], and the preference for salty tastes increases with increased smoking, alcohol intake, and obesity [30,31]. Therefore, unhealthy lifestyle factors such as smoking and alcohol intake might affect the preference for salt. Alcohol intake might be a consistent factor for excessive sodium intake because most Korean people eat salty foods while drinking alcohol, and these foods typically have a higher salt content than a usual meal [32]. 

In the present study, the sodium intake was 1.54 times higher in the group of subjects with both a current or previous history of smoking and current alcohol intake than in the group of subjects who were both never-smokers and non-drinkers (*p-interaction* = 0.02). Similarly, compared with non-smokers and non-drinkers, the preference for low-salt food decreased in the following order in a previous study: smoking only group, alcohol only group, and both smoking and alcohol group [15]. Exposure to both smoking and alcohol has a more serious impact on health than smoking or alcohol alone [33]. The correlations between current smoking and current drinking and between current smoking and hazardous drinking are statistically significant [34]. In addition, smokers tend to consume more alcohol than non-smokers [13,14]; drinking might lead to greater smoking, while smoking might encourage increased drinking. This bi-directional relationship might explain a high sodium intake with high alcohol intake [35].

Owing to the self-reported nature of the questionnaire, the present results might have underestimated smoking and alcohol consumption while overestimating other variables as a result of social desirability. In addition, because smoking dulls taste perception, a causal relationship with excessive sodium intake could not be explained in an earlier cross-sectional study [36]. However, a 24-h recall might be a more appropriate method than a food frequency questionnaire to consider ordinal associations, despite the inability of a 24-h recall to consider dietary patterns for sodium intake [37].

Despite these limitations, the 3-year nationwide KNHANES survey was conducted with representative subjects of the population; therefore, the results likely represent the general population. The reliability of the results is also high because the quality management was conducted in accordance with standard performance guidelines. Moreover, the use of three smoking factors increases the validity of the results. 

## 5. Conclusions 

Excessive sodium intake was higher with smoking experience and alcohol intake in Korean men, owing to a simultaneous effect of smoking and alcohol consumption. The main cause and route of excessive sodium intake identified in this study could be utilized as the primary basis of future measures for dietary sodium reduction policies and to identify high-risk groups. 
